# Sex differences in modifiable risk factors for stroke incidence and recurrence: the UCC-SMART study

**DOI:** 10.1007/s00415-024-12268-6

**Published:** 2024-03-16

**Authors:** Ina Rissanen, Maartje Basten, Lieza G. Exalto, Sanne A. E. Peters, Frank L. J. Visseren, Mirjam I. Geerlings, For the UCC-SMART-Study Group, M. J. Cramer, M. G. van der Meer, H. M. Nathoe, G. J. de Borst, M. L. Bots, M. I. Geerlings, M. H. Emmelot-Vonk, P. A. de Jong, A. T. Lely, N. P. van der Kaaij, L. J. Kappelle, Y. M. Ruigrok, M. C. Verhaar, J. A. N. Dorresteijn, F. L. J. Visseren

**Affiliations:** 1 Julius Center for Health Sciences and Primary Care, University Medical Center Utrecht and Utrecht University, Utrecht, The Netherlands; 2 Department of Health Sciences, Vrije Universiteit Amsterdam, Amsterdam, The Netherlands; 3 Department of Neurology, University Medical Center Utrecht and Utrecht University, Utrecht, The Netherlands; 4 The George Institute for Global Health, School of Public Health, Imperial College London, London, UK; 5 The George Institute for Global Health, University of New South Wales, Sydney, NSW Australia; 6 Department of Vascular Medicine, University Medical Center Utrecht, Utrecht, The Netherlands; 7 Department of General Practice, Amsterdam UMC, Location University of Amsterdam, Amsterdam, The Netherlands; 8 Amsterdam Public Health, Aging & Later Life, Amsterdam, The Netherlands; 9 Amsterdam Neuroscience, Neurodegeneration, and Mood, Anxiety, Psychosis, Stress, and Sleep, Amsterdam, The Netherlands; 10 Amsterdam Public Health, Mental Health program, Amsterdam, The Netherlands; 11 Amsterdam Public Health, Health Behaviors and Chronic Diseases program, Amsterdam, The Netherlands; 12 Amsterdam Public Health, Personalized Medicine, Amsterdam, The Netherlands

**Keywords:** Stroke, Sex differences, Risk factors, Cohort studies, Stroke recurrence

## Abstract

**Background and purpose:**

Risk factors for stroke differ between women and men in general populations. However, little is known about sex differences in secondary prevention. We investigated if sex interacted with modifiable risk factors for stroke in a large arterial disease cohort.

**Methods:**

Within the prospective UCC-SMART study, 13,898 patients (35% women) with atherosclerotic disease or high-risk factor profile were followed up to 23 years for stroke incidence or recurrence. Hypertension, smoking, diabetes, overweight, dyslipidemia, high alcohol use, and physical inactivity were studied as risk factors. Association between these factors and ischemic and hemorrhagic stroke incidence or recurrence was studied in women and men using Cox proportional hazard models and Poisson regression models. Women-to-men relative hazard ratios (RHR) and rate differences (RD) were estimated for each risk factor. Left-truncated age was used as timescale.

**Results:**

The age-adjusted stroke incidence rate was lower in women than men (3.9 vs 4.4 per 1000 person-years), as was the age-adjusted stroke recurrence rate (10.0 vs 11.7). Hypertension and smoking were associated with stroke risk in both sexes. HDL cholesterol was associated with lower stroke incidence in women but not in men (RHR 0.49; CI 0.27–0.88; and RD 1.39; CI − 1.31 to 4.10). Overweight was associated with a lower stroke recurrence in women but not in men (RHR 0.42; CI 0.23–0.80; and RD 9.05; CI 2.78–15.32).

**Conclusions:**

In high-risk population, sex modifies the association of HDL cholesterol on stroke incidence, and the association of overweight on stroke recurrence. Our findings highlight the importance of sex-specific secondary prevention.

**Supplementary Information:**

The online version contains supplementary material available at 10.1007/s00415-024-12268-6.

## Introduction

Stroke is the second largest cause of death and third largest cause of disability, accounting for 10% of the disease burden in the world [1]. Stroke results not only in physical disabilities but also in problems with cognition and overall functioning [2]. Stroke incidence is higher among men than women [3], but the sex difference attenuates with increasing age [4]. More than 90% of the stroke burden is attributable to modifiable risk factors, and three quarters of stroke burden is attributable to behavioral factors such as smoking, poor diet, and low physical activity [5]. Modifiable risk factors are the major focus of stroke primary and secondary prevention [6].

Stroke risk factors differ between sexes. Some risk factors are more prevalent among either women or men, whereas other factors differ to what extend they predict the stroke risk. Modifiable risk factors such as hypertension, smoking, overweight and dyslipidemia are more common among men [7, 8]. Previous studies have also found sex-specific risk factors for stroke such as gestational hypertension in women [9]. However, neither difference in prevalence of traditional risk factors nor existence of sex-specific risk factors fully explain the different stroke incidence and recurrence between sexes. It seems that the differences are partly due to different effects of modifiable risk factors in women and men [10]. For example, atrial fibrillation, hypertension, obesity, smoking, and type 2 diabetes are stronger risk factors for women [10, 11].

Most previous studies on sex differences in risk factor effects have studied stroke incidence in general populations, and little is known on secondary prevention. One of the strongest risk factors for stroke is previous history of stroke and other cardiovascular disease. Men have higher risk for a recurrent cardiovascular event, especially stroke, than women [11–13]. On the other hand, women are less likely to have optimal treatment for cardiovascular risk factors [14–16], suggesting an increased risk for recurrent events.

The aim of this study was to investigate sex differences in the prevalence of risk factors and effect modification by sex in patients with established cardiovascular disease or a high-risk factor profile. Sex differences were studied separately for stroke incidence in a high-risk patient cohort and for stroke recurrence. We investigate sex differences, categorized binary as women and men and assigned at birth based on biological attributes [17]. We do not distinguish the biological sex differences from socially constructed gender differences. We refer to all differences as ‘sex differences’ for clarity.

## Methods

### Study sample

Patient data from the Utrecht Cardiovascular Cohort—Second Manifestations of ARTerial disease (UCC-SMART) study were used, an ongoing single-center prospective cohort study at the University Medical Center (UMC) Utrecht, the Netherlands, on cardiovascular disease and their risk factors [18]. The study was designed with the aim of determining the prevalence of concomitant atherosclerotic disease and risk factors, as well as studying the incidence and prevalence of future cardiovascular events and their predictors. Starting from September 1996, patients aged 18–80 years referred for management of cardiovascular disease or severe risk factors in UMC Utrecht have been recruited. Patients with cerebrovascular disease, coronary artery disease, abdominal aortic aneurysm, peripheral artery disease, renal artery stenosis or one or more of the following cardiovascular risk factors, if rated as severe, were included: hypertension, hyperlipidemia, diabetes mellitus, renal insufficiency, and a positive family medical history. Risk factors were rated severe by physicians if, e.g., they were difficult to control or they had caused target organ damage. More detailed definitions of cardiovascular diseases and severe risk factors are described elsewhere [18]. If patients had a history of multiple vascular events or risk factors, the referral reason (usually the most recent event) was listed as the qualifying inclusion diagnosis and any comorbidities were also registered. Excluded were pregnant women, patients with a short life expectancy, and those insufficiently fluent in Dutch. Qualifying patients were recruited upon their first visit to the outpatient clinics and hospital wards of the departments of vascular medicine, internal medicine, nephrology, neurology, cardiology, cardiac surgery, obstetrics, and vascular surgery.

The SMART study was approved by the medical ethics committee of the UMC Utrecht, according to the guidelines of the Declaration of Helsinki of 1975. Written informed consent was obtained from all individuals included. For the current study, data from 13,898 participants enrolled between September 1996 and December 2019 were included.

### Previous arterial disease and risk factors

Definitions of enrollment diagnoses, risk factors, and departments of recruitment have been previously described [18]. At baseline, all patients underwent a standardized vascular screening protocol including a health questionnaire, physical examination, laboratory testing, ankle-branchial index, and an abdominal, aortic, and carotid ultrasound. Cardiovascular conditions and risk factors were ascertained in these baseline examinations.

History of cerebrovascular events at baseline was based on a composite score made of previous medical history of ischemic cerebrovascular disease or carotid artery surgery or angioplasty, or a physician diagnosis at study inclusion of one among the following conditions: transient ischemic attack (TIA), ischemic stroke, or ischemic retinal syndrome. Definition of ischemic stroke in inclusion was sudden onset and > 24 h of: (1) in carotid stroke temporary motor weakness in one half of the body, language disorder, blindness in one eye; (2) in vertebrobasilar stroke two or more simultaneously: bilateral motor weakness or paraesthesia, dizziness, diplopia, dysphagia, ataxia, dysarthria; (3) in stroke of unknown vascular region hemianopia, dysarthria. There was no necessity for an increase in the modified Rankin scale disability. Definition of TIA was as for ischemic stroke, but duration of ≤ 24 h. Definition of retinal ischemic syndrome was visual field defect diagnosed as retinal syndrome by an ophthalmologist. At the time of the study inclusion, symptomatic cerebrovascular disease was considered the main indication for carotid artery surgery or angioplasty, and hence history of them was considered as history of cerebrovascular events. Stroke incidence was studied among those without previous cerebrovascular event at baseline (i.e., high-risk patients), and stroke recurrence among those with previous cerebrovascular event at baseline.

We examined the following risk factors: hypertension, systolic blood pressure, diastolic blood pressure, current and former smoking, smoked pack-years, diabetes, overweight and obesity, hypercholesterolemia, LDL cholesterol, HDL cholesterol, current and former alcohol use, low and high alcohol use, and low physical activity. Office blood pressure measurements were performed with automated blood pressure monitors (Iso-Stabil5; Speidel & Keller, Germany) on the arm with the highest blood pressure. The mean of 3 measurements on that arm was recorded. Hypertension was defined as a mean systolic blood pressure of > 160 mmHg, a mean diastolic blood pressure of > 95 mmHg according to guidelines in 2001, or self-reported use of antihypertensive medication. Due to study selection criteria, nearly all participants had systolic blood pressure > 140 mmHg or diastolic blood pressure > 90 mmHg, and therefore, updated definition was not used. Antihypertensive medication included angiotensin-converting enzyme inhibitors, angiotensin II receptor blockers, beta-blockers, alpha-blockers, calcium antagonists, diuretics, aldosterone antagonists, central acting antihypertensives, and direct vasodilators.

Smoking habits were assessed with baseline questionnaires. Smoking habits were categorized as never, former, or current. Individuals who had quit smoking during the past year were considered current users. Pack-years were calculated by multiplying the number of packs of cigarettes smoked per day by the number of years the person had smoked.

An overnight fasting venous blood sample was taken at study inclusion to determine glucose levels. Type 2 diabetes mellitus was defined as either a referral or self-reported diagnosis, or a fasting plasma glucose ≥ 7 mmol/L with initiation of glucose-lowering treatment within 1 year, or baseline use of hypoglycemic agents or insulin. Height and weight were measured at baseline, and the BMI was calculated as kg/m^2^. Overweight was considered as BMI ≥ 25 to < 30, and obesity as BMI ≥ 30.

Lipid levels were determined at study inclusion from an overnight fasting venous blood sample. Total cholesterol and triglycerides were measured with a commercial enzymatic dry chemistry kit (Johnson & Johnson, New Brunswick, USA). High-density lipoprotein cholesterol (HDL) was measured with a commercial enzymatic kit (Boehringer, Germany) and low-density lipoprotein cholesterol (LDL) was calculated using the Friedewald formula up to triglyceride levels of 9 mmol/L to reduce missing values [19]. Hypercholesterolemia was considered if serum cholesterol was ≥ 6.2 mmol/L or person was using lipid-lowering medication. Lipid-lowering medication included use of statins, fibrates, bile acid sequestrants or nicotinic acid.

Alcohol intake and pattern of leisure-time physical activity were assessed with baseline questionnaires. Alcohol intake was categorized as never, former, or current. Individuals who had quit drinking during the past year were considered current users. Current alcohol use was considered low if a person drank less than one unit per week, and as high if a person drank more than ten units per week. Physical activity was asked as how many hours per week patients spent on leisure‐time physical activities, with focus on sport and other physical activities, excluding housekeeping and work‐related physical activity. Each activity was assigned a specific metabolic equivalent (MET) intensity derived from the Compendium of Physical Activity [20]. For each type of physical activity, the time spent on this activity was multiplied with MET intensity of the activity, expressed in MET hours per week. The total amount of physical activity per week was the sum of these values. Low physical activity was defined as < 20 MET hours per week.

As a baseline characteristic, use of antiplatelet or anticoagulant medication was recorded from the questionnaires and defined as self-reported use of either antiplatelet agents or vitamin K antagonists.

### Stroke incidence and recurrence

During follow-up, patients received questionnaires on hospital admissions and outpatient clinic visits twice a year to establish the incidence and recurrence of cardiovascular events, including strokes and fatal events [18]. If an event was reported, all relevant hospital documents, and laboratory and radiologic findings were collected. All events were audited independently by three physicians of the UCC-SMART endpoint committee. The outcomes for this study were incident or recurrent ischemic or hemorrhagic strokes whether fatal or not. Ischemic stroke was defined as > 24 h of associated clinical signs causing increased disability of ≥ 1 grade on modified Rankin scale, and new (hemorrhagic) infarction on CT or MRI < 2 weeks after stroke. Hemorrhagic stroke included cerebral hemorrhage or subarachnoid hemorrhage confirmed with either CT, MRI, or surgery. Strokes defined as > 24 h of associated clinical signs causing increased disability of ≥ 1 grade on modified Rankin scale, but no brain imaging performed, were included as undetermined strokes. In case of fatal strokes, the definition was cerebral infarction, cerebral hemorrhage, or stroke without radiological confirmation leading to Rankin score 4 or 5 followed by death (assuming that patient would not have died without stroke). Incident and recurrent strokes were studied separately, excluding those with/without previous strokes from the analyses.

### Statistical analyses

Cox proportional hazard models were used to estimate hazard ratios (HR) and 95% confidence intervals (95% CI) for stroke incidence or recurrence in total sample and separately in women and men. Left-truncated age (i.e., persons enter the follow-up at baseline age, not at birth) was used as timescale in Cox regression analyses [21]. Individual time at risk was measured from the entry age at inclusion until the stroke event or death, lost to follow-up, or January 1st, 2020, whichever came first. In total sample analyses, each risk factor, sex, and a product term of risk factor with sex was included to obtain the women-to-men relative hazard ratio (RHR) for that risk factor. For interpretability of the results, each risk factor was studied in a separate model and no further adjustments were made, as each risk factor was considered to have its own confounders and their sex-interactions. As sensitivity analyses (results shown in the Supplemental Material) multivariable Cox regression models with adjustments for potential confounders were conducted to explore the potential index event bias, including interaction terms between each confounder variable and sex.

We also estimated sex differences on the absolute scale as incidence and recurrence rates per 1000 person-years in women and men. The women-to-men difference of rate differences were estimated using Poisson regression models for categorical variables following the previously published tutorial. Individual time at risk was measured from the inclusion until the stroke event or death, loss to follow-up, or January 1st, 2020, whichever came first. Follow-up time was recorded as per 1000 person-years. The Poisson regression models were adjusted for age with an interaction term of age and sex (not shown).

Cox regression analyses were performed using R version 4.0.3 and Poisson regression analyses using Stata version 13SE.

## Results

### Characteristics of women and men

Of the total study sample of 13,898 patients, 4864 (35.0%) were women and 9034 (65.0%) were men. Mean follow-up time was 9.4 years (SD 5.8 years) ranging up to 23.3 years, and 1196 persons (8.6%) were lost to follow-up. In total, there were 131,106 person-years of follow-up. At baseline, 20% of women and 19% of men had a history of cerebrovascular events, and 35% of women and 62% of men had a history of other cardiovascular disease (Table 1). The remaining persons were included due to high cardiovascular risk factor profile including hypertension, dyslipidemia, and diabetes mellitus. Baseline characteristics of persons with and without previous history of cerebrovascular events are shown in Supplement Table 1.

**Table 1:** Baseline characteristics of the total sample as well as women and men

	Total (*N* = 13,898)	Women (*n* = 4864)	Men (*n* = 9034)	*P* value of sex difference
	Mean (SD) / *n* (%)	Mean (SD) / *n* (%)	Mean (SD) / *n* (%)	
**Age**	56.7 (12.5)	54.1 (13.8)	58.2 (11.5)	< 0.001
**Hypertension**				
Hypertension	7675 (56.6)	2861 (59.9)	4814 (54.7)	< 0.001
Systolic BP (mmHg)	140 (22)	140 (24)	141 (21)	0.503
Diastolic BP (mmHg)	83 (13)	82 (14)	83 (12)	0.016
Antihypertensive medication	6473 (47.8)	2416(50.7)	4057 (46.3)	< 0.001
**Smoking**				
Current smoking	3746 (27.1)	1240 (25.6)	2506 (27.9)	0.005
Former smoking	6016 (43.5)	1666 (34.4)	4350 (48.3)	< 0.001
Pack-years smoked	16.1 (18.8)	11.3 (15.9)	18.7 (19.6)	< 0.001
**Diabetes**				
Diabetes	2450 (17.6)	755 (15.5)	1695 (18.8)	< 0.001
Fasting glucose mmol/l	6.2 (1.9)	6.1 (1.9)	6.3 (1.9)	< 0.001
HbA1c (%)	5.9 (1.0)	5.9 (1.1)	5.9 (1.0)	0.001
**BMI**				
BMI	26.9 (4.4)	26.7 (5.2)	27.0 (4.0)	0.002
Overweight	6151 (44.5)	1714 (35.4)	4437 (49.4)	< 0.001
Obesity	2803 (20.2)	1115 (23.0)	1688 (18.7)	< 0.001
**Dyslipidemia**				
Hypercholesterolemia	10,452 (76.9)	3624 (76.1)	6828 (77.2)	0.142
Total cholesterol	5.1 (1.4)	5.4 (1.2)	4.9 (1.3)	< 0.001
LDL (mmol/L)	3.0 (1.2)	3.2 (1.2)	2.9 (1.1)	< 0.001
HDL (mmol/L)	1.3 (0.4)	1.5 (0.4)	1.2 (0.3)	< 0.001
Triglycerides (mmol/L)	1.8 (1.8)	1.6 (1.8)	1.8 (1.8)	< 0.001
Medication for dyslipidemia	6028 (44.7)	1720 (36.7)	4308 (49.0)	< 0.001
**Alcohol use**				
Current use	7990 (57.8)	2378 (49.3)	5612 (62.4)	< 0.001
Former alcohol use	3192 (23.1)	880 (18.2)	2312 (25.7)	< 0.001
Low alcohol use	4338 (31.6)	2404 (50.2)	1934 (21.6)	< 0.001
High alcohol use	3505 (25.5)	547 (11.4)	2958 (33.1)	< 0.001
**Physical activity**				
Total physical activity (MET h/week)	46.2 (41.8)	44.2 (40.5)	47.2 (42.5)	< 0.001
Sport activity (MET h/week)	7.8 (13.2)	6.5 (11.2)	8.4 (14.0)	< 0.001
Other activity (MET h/week)	39.3 (38.7)	38.7 (38.1)	39.6 (39.1)	0.203
Low activity	3994 (28.9)	1423 (29.4)	2571 (28.7)	0.337
**History of stroke**	2657 (19.1)	991 (20.4)	1666 (18.5)	0.006
**Other CVD history**	7284 (52.7)	1669 (34.6)	5615 (62.4)	< 0.001
**Anticoagulant or antiplatelet medication**	8182 (58.9)	2132 (43.8)	6050 (67.0)	< 0.001

Hypertension, diabetes, and hypercholesterolemia based on having either diagnosis or using medication for the condition. Overweight = BMI 25–29.99, obesity = BMI ≥ 30, *LDL* low-density lipoprotein, *HDL* high-density lipoprotein, low alcohol use = less than one unit per week among current users, high alcohol use = more than 10 units per week among current users, low physical activity =  < 20 metabolic equivalent (MET) hours per week. Difference between women and men tested with Pearson Chi-square test or independent samples *t* test

Table 1 shows the baseline characteristics of the total study sample stratified by sex. Women were on average younger than men (mean age 54 vs 58 years), and there were slight differences in the prevalence of risk factors. Women were less likely to receive anticoagulant or antiplatelet medication (44% vs 67%).

### Stroke incidence

During the 107,334 person-years of follow-up, 127 (3.3%) of 3873 women and 297 (4.0%) of 7368 men had an incident stroke. Table 2 shows as results of Cox regression analyses the HRs for stroke incidence per modifiable risk factor in women and men separately, as well as women-to-men RHRs. In women, hypertension (HR 1.74; 95% CI 1.15–2.65), current smoking (HR 2.05; 95% CI 1.33–3.14), and diabetes (HR 1.68; 95% CI 1.15–2.45) were associated with increased risk for stroke incidence. Furthermore, HDL (HR 0.51; 95% CI 0.32–0.81) and current alcohol use (HR 0.52; 95% CI 0.33–0.81) were associated with a lower risk for stroke incidence in women.

**Table 2:** Hazard ratios and women-to-men relative hazard ratios for stroke incidence per modifiable risk factor

	Women	Men	Women to men
	HR (95% CI)	HR (95% CI)	RHR (95% CI)
Hypertension			
Hypertension	1.74 (1.15–2.65)	1.38 (1.09–1.75)	1.24 (0.77–2.00)
Systolic BP per 10 mmHg	1.09 (1.02–1.17)	1.08(1.02–1.14)	1.01 (0.93–1.10)
Diastolic BP per 5 mmHg	1.14 (1.01–1.29)	1.08 (0.98–1.19)	1.06 (0.91–1.24)
Smoking			
Current smoking	2.05 (1.33–3.14)	2.03 (1.43–2.88)	1.01 (0.59–1.75)
Former smoking	1.03 (0.66–1.61)	1.16 (0.83–1.62)	0.89 (0.51–1.55)
Pack-years per 10 years	1.06 (0.96–1.17)	1.11 (1.05–1.17)	0.96 (0.85–1.07)
Diabetes			
Diabetes	1.68 (1.15–2.45)	1.19 (0.90–1.56)	1.41 (0.88–2.25)
BMI			
BMI per 1 kg/m^2^	1.01 (0.97–1-04)	0.97 (0.94–1.00)	1.04 (0.99–1.09)
Overweight	1.02 (0.68–1.53)	0.85 (0.67–1.10)	1.19 (0.74–1.92)
Obesity	1.09 (0.69–1.71)	0.74 (0.52–1.06)	1.47 (0.82–2.61)
Overweight or obesity	1.04 (0.73–1.50)	0.83 (0.65–1.05)	1.26 (0.81–1.94)
Dyslipidemia			
Hypercholesterolemia	1.04 (0.64–1.69)	1.03 (0.78–1.36)	0.99 (0.57–1.72)
LDL per 1 mmol/L	1.02 (0.89–1.17)	1.12 (1.01–1.25)	0.90 (0.76–1.08)
HDL per 1 mmol/L	0.51 (0.32–0.81)	1.03 (0.72–1.48)	0.49 (0.27–0.88)
Alcohol			
Current alcohol use	0.52 (0.33–0.81)	0.81 (0.57–1.15)	0.65 (0.37–1.16)
Former alcohol use	1.23 (0.82–1.85)	1.01 (0.70–1.45)	1.21 (0.70–2.10)
Low alcohol use	1.19 (0.81–1.75)	1.27 (0.94–1.70)	0.93 (0.57–1.52)
High alcohol use	0.79 (0.40–1.53)	1.15 (0.89–1.50)	0.68 (0.33–1.39)
Physical activity			
Low physical activity	1.32 (0.93–1.89)	1.42 (1.12–1.80)	0.92 (0.60–1.41)
Physical activity per 10 MET h/week	0.98 (0.94–1.03)	0.95 (0.92–0.98)	1.04 (0.98–1.10)

Each risk factor was studied in a separate Cox proportional hazard model. Left-truncated age used as timescale in models

*HR* hazard ratio, *CI* confidence interval, *RHR* relative hazard ratio

**Fig. 1:**
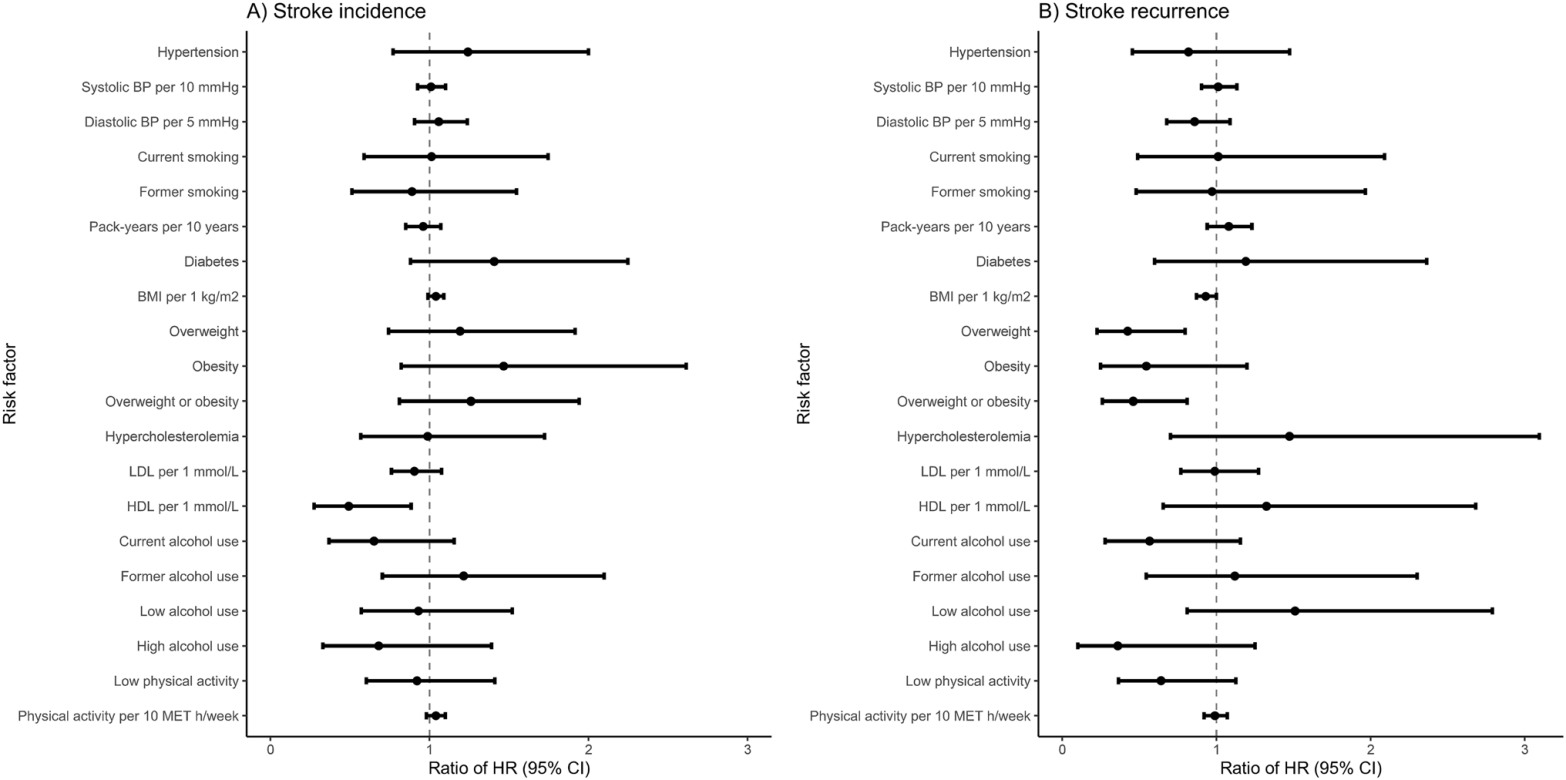
Women-to-men ratio of hazard ratio (95% CI) for stroke incidence (**A**) and for stroke recurrence (**B**) per risk factor

In men, hypertension (HR 1.38; 95% CI 1.09–1.75), current smoking (HR 2.03; 95% CI 1.43–2.88), LDL cholesterol (HR 1.12; 95% CI 1.01–1.25), and low physical activity (HR 1.42; 95% CI 1.12–1.80) were associated with an increased risk for stroke incidence. HDL cholesterol showed a women-to-men RHR of 0.49 (95% CI 0.27–0.88) indicating its association with risk for stroke incidence to be lower in women compared to men (Fig. 1). The finding was similar, even though did not stay statistically significant, after adjustment for other risk factors in sensitivity analyses (RHR 0.58; 95% CI 0.31–1.10) (Supplement Table 2).

Looking at incidence rate differences using Poisson regression models, we found that age-adjusted stroke incidence rates per 1000 person-years were 3.91 (95% CI 3.22–4.60) in women and 4.36 (95% CI 3.86–4.86) in men (Table 3). Incidence rate differences associated with risk factors were not significantly different between women and men.

**Table 3:** Stroke incidence rates for women and men and difference of incidence rate differences per risk factor

	Women (*n* = 4864)	Men (*n* = 9034)	Women to men
	*n*/1000 person-years	*n*/1000 person-years	Difference of rate differences (95% CI)
Stroke incidence	3.91 (3.22–4.60)	4.36 (3.86–4.86)	0.45 (− 0.40 to 1.30)
Hypertension			
Hypertension	4.62 (3.70–5.53)	4.89 (4.16–5.61)	− 0.80 (− 2.47 to 0.86)
Systolic > 140 mmHg	4.14 (3.18–5.09)	4.89 (4.12–5.66)	0.53 (− 1.20 to 2.26)
Diastolic > 90 mmHg	4.96 (3.45–6.48)	5.05 (3.89–6.21)	− 0.50 (− 2.62 to 1.62)
Smoking			
Current smoking	6.26 (4.37–8.16)	6.80 (5.40–8.20)	0.47 (− 2.23 to 3.16)
Former smoking	3.21 (2.18–4.25)	3.80 (3.18–4.42)	0.52 (− 1.30 to 2.33)
Pack-years > 20 years	4.81 (3.25–6.37)	5.11 (4.27–5.96)	0.10 (− 1.91 to 2.11)
Diabetes			
Diabetes	5.84 (3.98–7.69)	4.95 (3.76–6.15)	− 1.68 (− 4.07 to 0.70)
BMI			
Overweight	3.94 (2.83–5.04)	4.25 (3.57–4.94)	− 0.83 (− 2.76 to 1.11)
Obesity	4.11 (2.68–5.54)	3.65 (2.54–4.77)	− 1.60 (− 3.91 to 0.70)
Dyslipidemia			
Hypercholesterolemia	3.93 (3.18–4.69)	4.40 (3.82–4.98)	− 0.02 (− 2.15 to 2.11)
LDL > 2 mmol/L	4.06 (3.29–4.84)	4.64 (4.05–5.24)	− 0.11 (− 2.31 to 2.09)
HDL > 1 mmol/L	3.62 (2.91–4.33)	4.14 (3.54–4.74)	1.39 (− 1.31 to 4.10)
Alcohol			
Current alcohol use	2.18 (1.38–2.98)	3.72 (3.13–4.32)	1.06 (− 1.14 to 3.27)
Former alcohol use	5.77 (4.00–7.53)	5.34 (4.30–6.38)	− 0.91 (− 3.74 to 1.93)
Low alcohol use	2.76 (1.13–4.39)	4.53 (3.65–5.42)	1.69 (− 0.59 to 3.96)
High alcohol use	4.20 (3.20–5.20)	4.94 (3.77–6.10)	0.66 (− 1.39 to 2.67)
Physical activity			
Low physical activity	4.78 (3.51–6.04)	5.74 (4.66–6.82)	0.59 (− 1.32 to 2.51)

Each risk factor was studied in a separate Poisson regression model adjusted for age with an interaction term of sex and age

*CI* confidence interval

### Stroke recurrence

**Fig. 2:**
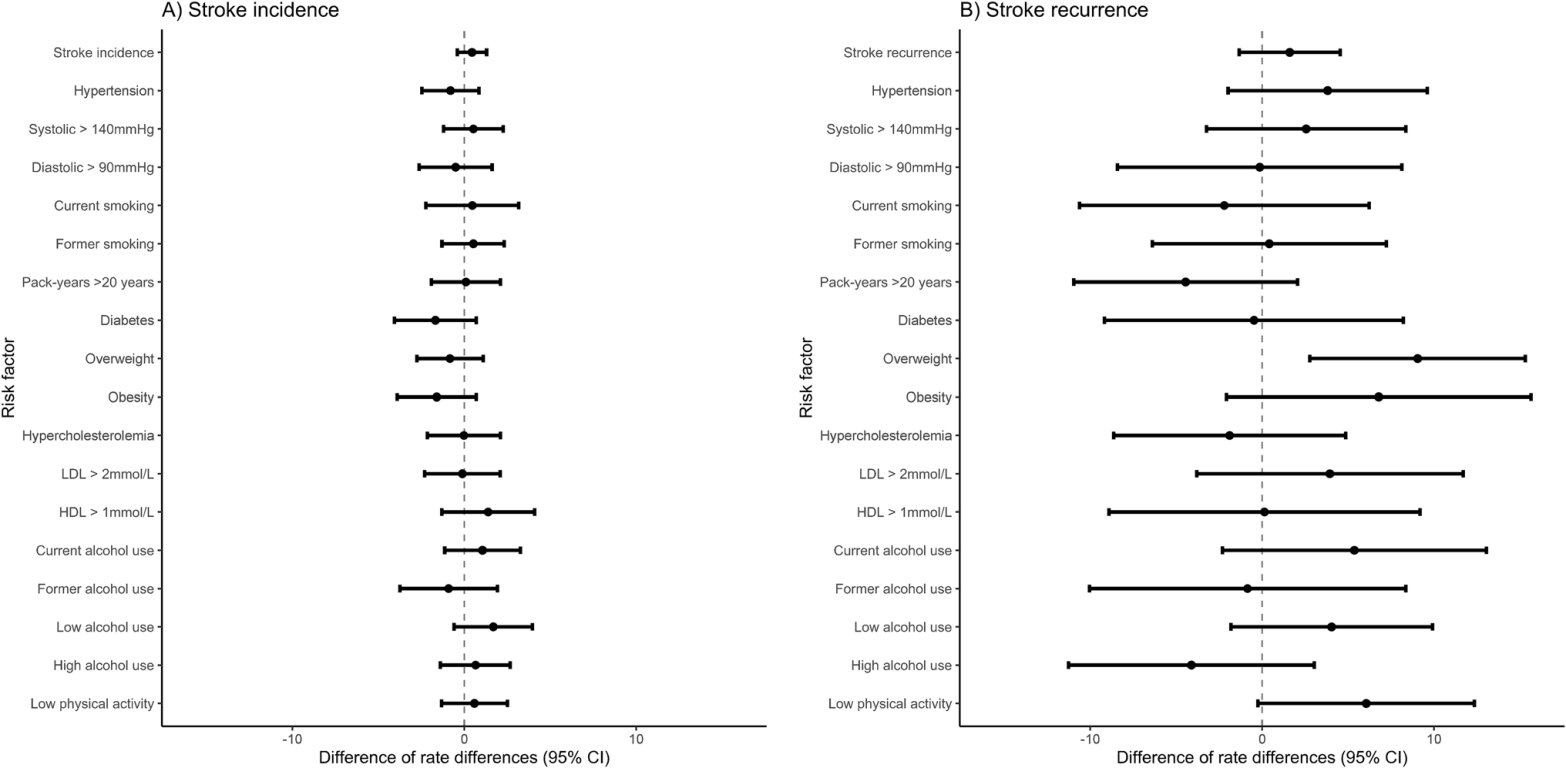
Women-to-men difference of incidence rate differences (95% CI) for stroke incidence (**A**) and for stroke recurrence (**B**) per risk factor

During the 23,772 person-years of follow-up, 76 (7.7%) of 991 women and 171 (10.3%) of 1666 men had a recurrent ischemic stroke. Table 4 shows the Cox regression results for stroke recurrence per modifiable risk factors in women and men separately, as well as women-to-men RHRs. In women, overweight (0.48; 95% CI 0.28–0.82) was associated with a lower risk for stroke recurrence. In men, hypertension (HR 1.73; 95% CI 1.24–2.41) and low physical activity (HR 1.64; 95% CI 1.21–2.21) were associated with an increased risk for stroke recurrence. Overweight showed a women-to-men RHR of 0.42 (95% CI 0.23–0.80) indicating its association with risk for stroke recurrence to be lower in women compared to men (Fig. 2). The finding persisted after adjustment for other risk factors (RHR 0.42; 95% CI 0.22–0.79) (Supplement Table 3).

**Table 4:** Hazard ratios and women-to-men relative hazard ratios for stroke recurrence per modifiable risk factor

	Women	Men	Women to men
	HR (95% CI)	HR (95% CI)	RHR (95% CI)
Hypertension			
Hypertension	1.29 (0.79–2.12)	1.73 (1.24–2.41)	0.82 (0.45–1.47)
Systolic BP per 10 mmHg	1.10 (0.99–1.21)	1.13 (1.06–1.22)	1.01 (0.90–1.13)
Diastolic BP per 5 mmHg	0.93 (0.76–1.14)	1.06 (0.93–1.22)	0.86 (0.68–1.09)
Smoking			
Current smoking	1.51 (0.84–2.71)	1.25 (0.79–1.97)	1.01 (0.49–2.09)
Former smoking	1.09 (0.62–1.93)	1.15 (0.75–1.77)	0.97 (0.48–1.97)
Pack-years per 10 years	1.11 (0.99–1.24)	1.03 (0.96–1.11)	1.08 (0.94–1.23)
Diabetes			
Diabetes	1.37 (0.76–2.47)	1.29 (0.89–1.86)	1.19 (0.60–2.36)
BMI			
BMI per 1 kg/m^2^	0.94 (0.89–1.00)	1.01 (0.97–1.06)	0.93 (0.87–1.00)
Overweight	0.48 (0.28–0.82)	1.20 (0.86–1.68)	0.42 (0.23–0.80)
Obesity	0.70 (0.38–1.31)	1.21 (0.74–1.97)	0.55 (0.25–1.20)
Overweight or obesity	0.55 (0.34–0.86)	1.21 (0.87–1.68)	0.46 (0.26–0.81)
Dyslipidemia			
Hypercholesterolemia	1.38 (0.72–2.66)	1.03 (0.71–1.48)	1.47 (0.70–3.10)
LDL per 1 mmol/L	1.06 (0.86–1.30)	1.10 (0.94–1.27)	0.99 (0.77–1.27)
HDL per 1 mmol/L	0.83 (0.48–1.42)	0.64 (0.41–1.02)	1.32 (0.65–2.68)
Alcohol			
Current alcohol use	0.54 (0.31–0.93)	0.91 (0.57–1.43)	0.57 (0.28–1.16)
Former alcohol use	1.04 (0.60–1.80)	0.96 (0.60–1.53)	1.12 (0.54–2.30)
Low alcohol use	1.52 (0.94–2.46)	1.01 (0.69–1.48)	1.51 (0.81–2.79)
High alcohol use	0.29 (0.09–0.95)	0.79 (0.56–1.13)	0.36 (0.10–1.25)
Physical activity			
Low physical activity	0.99 (0.61–1.60)	1.64 (1.21–2.21)	0.64 (0.36–1.13)
Physical activity per 10 MET h/week	0.99 (0.93–1.05)	0.99 (0.95–1.03)	0.99 (0.92–1.07)

Each risk factor was studied in a separate Cox proportional hazard model. Left-truncated age used as timescale in models

*HR* hazard ratio, *CI* confidence interval, *RHR* relative hazard ratio

In Poisson regression models, age-adjusted recurrence rates of stroke per 1000 person-years were 10.04 (95% CI 7.68–12.40) in women and 11.65 (95% CI 9.90–13.39) in men (Table 5). The sex difference of stroke recurrence rate differences was 9.05 (95% CI 2.78–15.32) for overweight, indicating that overweight is associated with a lower recurrence rate in women than in men.

**Table 5:** Stroke recurrence rates for women and men and difference of incidence rate differences per risk factor

	Women (*n* = 4864)	Men (*n* = 9034)	Women to men
	*n*/1000 person-years	*n*/1000 person-years	Difference of rate differences (95% CI)
Stroke recurrence	10.04 (7.68–12.40)	11.65 (9.90–13.39)	1.61 (− 1.33 to 4.54)
Hypertension			
Hypertension	10.78 (7.81–13.75)	14.09 (11.57–16.61)	3.82 (− 1.98 to 9.62)
Systolic > 140 mmHg	11.54 (8.09–14.99)	14.35 (11.65–17.06)	2.57 (− 3.24 to 8.37)
Diastolic > 90 mmHg	13.03 (6.45–19.62)	14.30 (10.16–18.43)	− 0.14 (− 8.42 to 8.14)
Smoking			
Current smoking	13.43 (7.89–18.97)	12.30 (9.40–16.59)	− 2.20 (− 10.63 to 6.23)
Former smoking	9.19 (5.69–12.69)	11.38 (8.98–13.78)	0.42 (− 6.39 to 7.23)
Pack-years > 20 years	13.11 (8.14–18.08)	11.56 (8.94–14.18)	− 4.45 (− 10.96 to 2.06)
Diabetes			
Diabetes	12.86 (6.12–19.59)	13.99 (9.46–18.53)	− 0.47 (− 9.17 to 8.22)
BMI			
Overweight	6.63 (4.71–9.55)	12.28 (9.78–14.78)	9.05 (2.78 to 15.32)
Obesity	9.69 (4.34–15.05)	13.09 (7.94–18.24)	6.79 (− 2.07 to 15.66)
Dyslipidemia			
Hypercholesterolemia	10.29 (7.69–12.89)	11.87 (9.85–13.90)	− 1.89 (− 8.64 to 4.86)
LDL > 2 mmol/L	9.68 (7.12–12.24)	11.97 (9.97–13.97)	3.94 (− 3.81 to 11.70)
HDL > 1 mmol/L	9.71 (7.29–12.15)	11.00 (8.93–13.08)	0.14 (− 8.92 to 9.20)
Alcohol			
Current alcohol use	6.43 (3.76–9.11)	11.19 (8.75–13.62)	5.37 (− 2.31 to 13.06)
Former alcohol use	13.47 (7.92–19.02)	12.00 (8.75–13.62)	− 0.85 (− 10.05 to 8.36)
Low alcohol use	2.47 (− 0.33–5.26)	9.94 (7.26–12.63)	4.05 (− 1.81 to 9.92)
High alcohol use	13.71 (7.26–12.63)	13.02 (8.98–17.06)	− 4.11 (− 11.27 to 3.04)
Physical activity			
Low physical activity	10.32 (6.34–14.31)	15.92 (12.41–19.43)	6.06 (− 0.24 to 12.36)

Each risk factor was studied in a separate Poisson regression model adjusted for age with an interaction term of sex and age

*CI* confidence interval

## Discussion

This secondary prevention study, including more than 13,000 persons with cardiovascular disease, examined sex differences in modifiable risk factors for ischemic and hemorrhagic stroke incidence and recurrence during up to 23 years of follow-up. We found that hypertension, smoking, and diabetes were associated with an increased risk for stroke incidence in women, and hypertension, smoking, LDL cholesterol and low physical activity with an increased risk for stroke incidence in men. Sex modified the effect of HDL cholesterol, which was associated with lower stroke incidence in women, but not in men. Hypertension and low physical activity were associated with risk for stroke recurrence in men. Sex modified the effect of overweight, which was associated with lower stroke recurrence in women, but not in men.

Previous studies in general populations have found sex differences in modifiable risk factors for stroke incidence [10, 22–25]. Women seem to have higher excess risk of stroke incidence associated with diabetes, obesity, hypertension, and smoking [10, 26, 27]. In contrast, some studies have found the association of stroke incidence and hypertension, obesity, and dyslipidemia to be similar in both sexes [23]. In this secondary prevention study in a cardiovascular disease population, we did not observe sex differences in risk factor associations for hypertension or smoking. However, HDL cholesterol showed association with lower risk for stroke incidence in women but not in men. Previous research suggests HDL function to be different between women and men, mainly because of sex hormones, which may explain our finding [28]. Of note, since this is the first study to report this, it needs to be replicated before firm conclusions can be drawn.

Risk factors of secondary cardiovascular events are different to those of primary events [29, 30]. Here, the study sample consisted of persons with cardiovascular disease, and we also studied stroke recurrence separately. Few previous studies on sex differences in risk factors for stroke recurrence suggest hypertension, dyslipidemia and diabetes to be risk factors for stroke recurrence in both sexes [13], and smoking and alcohol consumption to be risk factors in men [31]. We observed that hypertension and low physical activity were associated with increased stroke recurrence in men. Furthermore, overweight was associated with a decreased stroke recurrence in women but not in men. While being counterintuitive, also previous studies have reported overweight to associate with lower risk for stroke recurrence [32]. The ‘obesity paradox’ is usually explained by the protective effect of adipose tissue affecting the inflammatory mechanisms in stroke [33]. On the other hand, such a finding could be explained by index event bias [34]. As UCC-SMART cohort was selected based on the occurrence of cardiovascular event, the selection influenced the distribution of risk factors and the association of these risk factors with stroke recurrence. To control for index event bias, we conducted sensitivity analyses adjusting for other risk factors, and the finding of sex difference persisted. Overall, our findings suggest the association between obesity and stroke recurrence to be different between women and men; however, there could be several different mechanisms for such a finding. Further studies are warranted to explore the sex differences in ‘obesity paradox’ in other populations minimizing the collider bias.

It has been discussed that risk factors for stroke could differ between sexes due to biological mechanisms or to be related to differences in social determinants, risk behaviors, and risk factor treatments between women and men. Concerning biological factors, previous literature has suggested that sex hormones could play a role in effect modification of risk factors [22]. It has been shown that the effect of sex on stroke risk varies with age [35]. In this study, we took the effect of age into account using left-capped age as time scale in the Cox proportional hazard regression analyses and by adjusting for age*sex interaction in Poisson regression analyses. However, subgroup analyses in specific age-groups were not conducted.

In addition to biological mechanisms, gender is a wider social concept that shapes cardiovascular health during the life-course together with biological sex and socioeconomic position [36]. Here, we found that presence of hypertension and dyslipidemia were higher among women than men, but women were less likely to have medication for dyslipidemia at baseline. Of women with dyslipidemia, 47% had medication, compared to 63% of men. There was no difference in antihypertensive medication: 84% in women and men. Previous literature has shown that women are less likely to be prescribed antiplatelet, anticoagulant or antihypertensive medication as a measure of secondary prevention [16, 37]. In addition, women are also less likely than men to have treated and adequately controlled dyslipidemia [8, 38]. Unfortunately, in our sample, the use of medications was only recorded at baseline. Any interventions or changes in treatments during the follow-up were not considered as we did not have reliable information about this, which may have affected the findings.

### Strengths and limitations

Strengths of the present study include the prospective cohort study design reflecting clinical practice of patients with cardiovascular disease or high-risk factor profile being treated according to current national guidelines. Other strengths are the large number of individuals included, the substantial follow-up duration up to 23 years, and the large number of validated and clinically relevant stroke outcomes. In addition, only 8.6% of study sample was lost to follow-up. Most previous studies have investigated sex-related differences in stroke risk factors only in relative risk without investigating absolute risk. However, to design sex-specific health interventions for stroke risk factors, it is important to know the absolute risk increase [39].

The current study has some limitations. Women included in the sample were a specific selection of women with cardiovascular disease given their relatively young age and that they were referred to secondary health care for the treatment of their cardiovascular disease. Second, numbers of incident and recurrent stroke were relatively low. Third, we studied each risk factor individually; however, several risk factors may co-occur. Co-occurrence of risk factors affects the treatment decisions of high-risk individuals which may further affect our findings. Although we conducted multivariable modes as sensitivity analyses, we cannot fully rule out index event bias. Fourth, only baseline levels of risk factors and medication use were considered, and changes over time were not taken into account. Fifth, etiologies of first cardiovascular event and stroke incidence or recurrence were not considered. Finally, as this was a single-center study, and recruitment of participants was restricted to specific hospital wards, potential referral bias might affect the generalizability of the findings.

## Conclusions

In this high-risk population, risk factors for ischemic and hemorrhagic stroke incidence and recurrence differed between women and men. Sex modified the association of HDL cholesterol on stroke incidence, and the association of overweight on stroke recurrence. Our findings underline the potential differential effect of modifiable risk factors in stroke in women and men. Guidelines should include sex-specific recommendations for secondary prevention to raise awareness of sex differences among clinicians.

### Supplementary Information

Below is the link to the electronic supplementary material.

Supplementary file1 (DOCX 26 KB)

## Data Availability

Data are available upon reasonable request. The UCC-SMART Study group directs the academic focus of research using the UCC-SMART data and consists of members from both epidemiological and clinical cardiovascular research. Datasets are provided to interested researchers after approval of request by the UCC-SMART Study group. Access to the data request module can be applied for via ucc-smart@umcutrecht.nl.
