# Supplementary material for: Sex differences in modifiable risk factors for stroke incidence and recurrence: the UCC-SMART study
Source: J Neurol. 2024 Mar 16;271(6):3347–58. doi: 10.1007/s00415-024-12268-6 (PMC11136802; doi:10.1007/s00415-024-12268-6)
Supplement: Supplementary file 1 — Supplementary file1 (DOCX 26 KB) [file 415_2024_12268_MOESM1_ESM.docx]

**Supplemental Material**

Supplement Table 1. Basic characteristics of participants with and without baseline history of cerebrovascular events.

|  | History of cerebrovascular events (N=2657) | No history of cerebrovascular events (N=11229) |
| --- | --- | --- |
|  | Mean (SD) / n (%) | Mean (SD) / n (%) |
| **Female sex** | 991 (37.3) | 3871 (34.5) |
| **Age** | 60.0 (11.2) | 56.0 (12.6) |
| **Hypertension** |  |  |
| Hypertension | 1569 (69.5) | 6097 (55.6) |
| Systolic BP (mmHg) | 141 (22) | 140 (22) |
| Diastolic BP (mmHg) | 82 (12) | 83 (13) |
| Antihypertensive medication | 1339 (51.8) | 5125 (46.9) |
| **Smoking** |  |  |
| Current smoking | 823 (31.1) | 2920 (26.1) |
| Former smoking | 1195 (45.1) | 4815 (43.1) |
| Pack-years smoked | 19.0 (20.1) | 15.4 (18.3) |
| **Diabetes** |  |  |
| Diabetes | 422 (15.9) | 2025 (18.9) |
| Fasting glucose | 6.1 (1.6) | 6.3 (2.0) |
| HbA1c (%) | 5.8 (0.8) | 5.9 (1.1) |
| **BMI** |  |  |
| BMI | 26.6 (4.2) | 27.0 (4.5) |
| Overweight | 1186 (44.8) | 4959 (44.4) |
| Obesity | 456 (17.2) | 2343 (20.9) |
| **Dyslipidemia** |  |  |
| Hypercholesterolemia | 1998 (76.8) | 8446 (76.9) |
| Total cholesterol | 4.9 (1.2) | 5.1 (1.4) |
| LDL (mmol/L) | 2.9 (1.1) | 3.1 (1.2) |
| HDL (mmol/L) | 1.3 (0.4) | 1.3 (0.4) |
| Triglycerides (mmol/L) | 1.6 (1.2) | 1.8 (1.9) |
| Medication for dyslipidemia | 1242 (48.0) | 4779 (43.9) |
| **Alcohol use** |  |  |
| Alcohol current use | 1395 (52.7) | 6587 (59.0) |
| Former alcohol use | 733 (27.7) | 2456 (22.0) |
| Low alcohol use | 811 (30.8) | 3525 (31.8) |
| High alcohol use | 720 (27.4) | 2781 (25.1) |
| **Physical activity** |  |  |
| Total physical activity (MET h/week) | 43.9 (41.2) | 46.7 (42.0) |
| Sport activity (MET h/week) | 6.8 (12.4) | 8.0 (13.3) |
| Other activity (MET h/week) | 38.0 (38.4) | 39.6 (38.8) |
| Low activity | 829 (31.4) | 3162 (28.4) |
| **Anticoagulant or antiplatelet medication** | 2142 (80.6) | 6030 (53.7) |

Hypertension, diabetes, and hypercholesterolemia based on having either diagnosis or using medication for the condition. Overweight = BMI 25-29.99, obesity = BMI ≥ 30, LDL = low-density lipoprotein, HDL = high-density lipoprotein, low alcohol use = less than one unit per week, high alcohol use = more than 10 units per week, low physical activity = <20 metabolic equivalent (MET) hours per week. Difference between persons with and without cerebrovascular event history tested with Pearson chi-square test or independent samples t-test.

Supplement Table 2.

Hazard ratios and women-to-men relative hazard ratios for stroke incidence per modifiable risk factor adjusted for potential confounders.

|  | Women | Men | Women to men |
| --- | --- | --- | --- |
|  | HR (95% CI) | HR (95% CI) | RHR (95% CI) |
| **Hypertension** |  |  |  |
| Hypertension | 1.87 (1.20-2.90) | 1.51 (1.18-1.93) | 1.21 (0.73-1.99) |
| Systolic BP per 10mmHg | 1.10 (1.02-1.18) | 1.08 (1.02-1.14) | 1.01 (0.92-1.10) |
| Diastolic BP per 5mmHg | 1.15 (1.01-1.31) | 1.12 (1.01-1.23) | 1.03 (0.87-1.21) |
| **Smoking** |  |  |  |
| Current smoking | 2.03 (1.30-3.17) | 1.95 (1.36-2.78) | 1.06 (0.60-1.86) |
| Former smoking | 1.16 (0.73-1.84) | 1.21 (0.86-1.70) | 0.95 (0.54-1.68) |
| Pack-years per 10 years | 1.96 (0.95-1.17) | 1.11 (1.05-1.17) | 0.95 (0.85-1.07) |
| **Diabetes** |  |  |  |
| Diabetes | 1.49 (0.98-2.29) | 1.23 (0.92-1.63) | 1.22 (0.73-2.03) |
| **BMI** |  |  |  |
| BMI per 1 kg/m^2^ | 1.01 (0.97-1.05) | 0.97 (0.94-1.01) | 1.04 (0.99-1.09) |
| Overweight | 1.06 (0.70-1.61) | 0.87 (0.68-1.13) | 1.20 (0.73-1.95) |
| Obesity | 1.14 (0.72-1.82) | 0.78 (0.54-1.13) | 1.47 (0.81-2.66) |
| Overweight or obesity | 1.09 (0.75-1.59) | 0.85 (0.67-1.09) | 1.27 (0.81-1.99) |
| **Dyslipidemia** |  |  |  |
| Hypercholesterolemia | 0.97 (0.60-1.59) | 0.92 (0.69-1.22) | 1.03 (0.58-1.79) |
| LDL per 1 mmol/L | 1.03 (0.88-1.20) | 1.08 (0.96-1.21) | 0.95 (0.79-1.16) |
| HDL per 1 mmol/L | 0.62 (0.37-1.04) | 1.06 (0.72-1.55) | 0.58 (0.31-1.10) |
| **Alcohol** |  |  |  |
| Current alcohol use | 0.48 (0.30-0.78) | 0.88 (0.61-1.26) | 0.56 (0.31-1.03) |
| Former alcohol use | 1.15 (0.76-1.76) | 0.92 (0.64-1.34) | 1.24 (0.70-2.18) |
| Low alcohol use | 1.25 (0.83-1.87) | 1.23 (0.91-1.66) | 1.01 (0.61-1.66) |
| High alcohol use | 0.75 (0.39-1.48) | 1.09 (0.84-1.42) | 0.69 (0.34-1.42) |
| **Physical activity** |  |  |  |
| Low physical activity | 1.03 (0.71-1.51) | 1.29 (1.00-1.67) | 0.79 (0.50-1.24) |
| Physical activity per 10 MET h/week | 1.02 (0.97-1.07) | 0.96 (0.93-0.99) | 1.06 (1.00-1.13) |

HR = hazard ratio, CI = confidence interval, RHR = relative hazard ratio. Each risk factor was studied in a separate Cox proportional hazard model. Left-truncated age used as timescale in models. Hypertension was adjusted for smoking status, diabetes, BMI, hypercholesterolemia, alcohol use, and physical activity; smoking status and pack-years were adjusted for BMI, alcohol use, and physical activity (hypertension, diabetes, and dyslipidemia were considered mediating factors); diabetes was adjusted for hypertension, smoking status, BMI, hypercholesterolemia, alcohol use, and physical activity; BMI, overweight, and obesity were adjusted for smoking status, alcohol use, and physical activity (hypertension, diabetes and dyslipidemia were considered mediating factors); hypercholesterolemia and cholesterol levels were adjusted for hypertension, smoking status, diabetes, BMI, alcohol use, and physical activity; alcohol use was adjusted for smoking status, BMI, and physical activity (hypertension, diabetes, and dyslipidemia were considered mediating factors); and physical activity was adjusted for smoking status and alcohol use (hypertension, diabetes, BMI, and dyslipidemia were considered mediating factors). Multivariable models included interaction terms between each variable and sex.

Supplement Table 3. Hazard ratios and women-to-men relative hazard ratios for stroke recurrence per modifiable risk factor adjusted for potential confounders.

|  | Women | Men | Women to men |
| --- | --- | --- | --- |
|  | HR (95%CI) | HR (95%CI) | RHR (95%CI) |
| **Hypertension** |  |  |  |
| Hypertension | 1.48 (0.88-2.49) | 1.76 (1.25-2.50) | 0.88 (0.47-1.64) |
| Systolic BP per 10mmHg | 1.11 (1.01-1.23) | 1.13 (1.05-1.21) | 1.02 (0.91-1.15) |
| Diastolic BP per 5mmHg | 1.02 (0.83-1.25) | 1.07 (0.93-1.22) | 0.93 (0.73-1.19) |
| **Smoking** |  |  |  |
| Current smoking | 1.38 (0.78-2.54) | 1.31 (0.82-2.09) | 0.88 (0.42-1.85) |
| Former smoking | 1.16 (0.65-2.07) | 1.19 (0.77-1.85) | 1.00 (0.49-2.05) |
| Pack-years per 10 years | 1.11 (0.98-1.25) | 1.03 (0.96-1.11) | 1.07 (0.93-1.23) |
| **Diabetes** |  |  |  |
| Diabetes | 1.33 (0.70-2.51) | 1.14 (0.78-1.67) | 1.26 (0.60-2.63) |
| **BMI** |  |  |  |
| BMI per 1 kg/m^2^ | 0.95 (0.89-1.00) | 1.01 (0.97-1.06) | 0.93 (0.87-1.00) |
| Overweight | 0.47 (0.28-0.81) | 1.19 (0.85-1.68) | 0.42 (0.22-0.79) |
| Obesity | 0.68 (0.36-1.27) | 1.21 (0.74-1.97) | 0.53 (0.24-1.17) |
| Overweight or obesity | 0.53 (0.33-0.85) | 1.21 (0.87-1.67) | 0.45 (0.26-0.80) |
| **Dyslipidemia** |  |  |  |
| Hypercholesterolemia | 1.31 (0.67-2.56) | 0.93 (0.64-1.35) | 1.56 (0.73-3.32) |
| LDL per 1 mmol/L | 1.02 (0.83-1.27) | 1.13 (0.96-1.33) | 0.93 (0.71-1.21) |
| HDL per 1 mmol/L | 0.81 (0.45-1.48) | 0.68 (0.42-1.13) | 1.23 (0.57-2.68) |
| **Alcohol** |  |  |  |
| Current alcohol use | 0.48 (0.27-0.86) | 0.90 (0.57-1.43) | 0.53 (0.25-1.11) |
| Former alcohol use | 0.97 (0.56-1.70) | 0.92 (0.57-1.48) | 1.11 (0.53-2.30) |
| Low alcohol use | 1.67 (1.00-2.76) | 1.02 (0.69-1.51) | 1.57 (0.84-2.96) |
| High alcohol use | 0.26 (0.08-0.88) | 0.77 (0.54-1.11) | 0.34 (0.10-1.19) |
| **Physical activity** |  |  |  |
| Low physical activity | 0.75 (0.45-1.24) | 1.71 (1.22-2.38) | 0.47 (0.26-0.86) |
| Physical activity per 10 MET h/week | 1.02 (0.96-1.09) | 0.99 (0.95-1.03) | 1.02 (0.95-1.10) |

HR = hazard ratio, CI = confidence interval, RHR = relative hazard ratio. Each risk factor was studied in a separate Cox proportional hazard model. Left-truncated age used as timescale in models. Hypertension was adjusted for smoking status, diabetes, BMI, hypercholesterolemia, alcohol use, and physical activity; smoking status and pack-years were adjusted for BMI, alcohol use, and physical activity (hypertension, diabetes, and dyslipidemia were considered mediating factors); diabetes was adjusted for hypertension, smoking status, BMI, hypercholesterolemia, alcohol use, and physical activity; BMI, overweight, and obesity were adjusted for smoking status, alcohol use, and physical activity (hypertension, diabetes and dyslipidemia were considered mediating factors); hypercholesterolemia and cholesterol levels were adjusted for hypertension, smoking status, diabetes, BMI, alcohol use, and physical activity; alcohol use was adjusted for smoking status, BMI, and physical activity (hypertension, diabetes, and dyslipidemia were considered mediating factors); and physical activity was adjusted for smoking status and alcohol use (hypertension, diabetes, BMI, and dyslipidemia were considered mediating factors). Multivariable models included interaction terms between each variable and sex.
